# Vision Loss in a Healthy Child: A Case of Vitamin A Deficiency

**DOI:** 10.7759/cureus.31683

**Published:** 2022-11-19

**Authors:** Nicholas J Garza, Rafael Gonzalez, Jennifer Snider

**Affiliations:** 1 Pediatrics, Valley Children’s Healthcare, Madera, USA; 2 Pediatric Gastroenterology, Stanford Health Care, Palo Alto, USA

**Keywords:** night blindness, restrictive eating, xerosis, vision changes, cultural considerations, hmong culture, bitot’s spots, vitamin a deficiency

## Abstract

Vitamin A deficiency (VAD) is common in developing countries but rare in resource-rich countries. In developed countries, malabsorption and behavioral issues are more common reasons for VAD. The current case is an example of a healthy child who developed ocular symptoms due to vitamin A deficiency in the setting of cultural influences and emotional stressors.

## Introduction

Vitamin A, also known as retinol, is an essential fat-soluble vitamin that plays a crucial role in several physiologic processes including vision, immunity, cellular signaling, and reproduction [1]. Globally, vitamin A deficiency (VAD) is one of the leading causes of preventable blindness and affects up to 30% of children under five years old [2]. In developed countries, VAD is rare and is primarily related to an underlying medical condition (i.e., malabsorption) or behavioral conditions resulting in restrictive eating behaviors (i.e., anorexia and autism) [3,4]. As an essential micronutrient, vitamin A cannot be synthesized by the human body and needs to be obtained from dietary sources [5]. Vitamin A-rich foods include leafy green vegetables, carrots, tomatoes, mangoes, and sweet potatoes [6]. VAD can lead to a weakened immune system, anemia, visual disturbances, and blindness [5]. Xerophthalmia is the term for serum retinol concentrations <0.35 µmol/L [5]. Xerophthalmia commonly leads to night blindness, as well as corneal and conjunctival xerosis. If allowed to progress, VAD can ultimately lead to atrophic changes, known as keratomalacia, and blindness [1]. 

Our patient was evaluated for worsening eye nodules with complaints of visual changes. He was seen by several providers and diagnosed with different ophthalmologic conditions until he was discovered to have VAD. What makes this case unique is that it was not the result of either malabsorption or a developmental condition. The occurrence of VAD in this previously healthy and developmentally appropriate child was the result of cultural and social dynamics that shaped his environment. This case emphasizes that although presenting symptoms and physical exam findings are important aspects in medicine, obtaining a thorough history is a crucial component that can help guide evaluation and management. 

## Case presentation

Our patient is a previously healthy eight-year-old Hmong male who presented to the emergency department with a chief complaint of vision loss and red tender bumps around his eyes. His physical exam was significant for 3-millimeter erythematous papules on his lower eyelids bilaterally. The history of the presentation was gathered from chart review and with the use of a Hmong interpreter since the mother spoke some English but it was not her primary language. Four months prior to his presentation, he began to have photophobia. At that time, the patient was diagnosed with styes and discharged home with warm compresses. At his initial primary care follow-up, he was referred to an ophthalmologist who prescribed Polytrim eye drops. Over the next several months, he continued to be evaluated by multiple ophthalmologists who again diagnosed him with styes and prescribed prednisolone eye drops. During this time, symptoms continued to worsen, and he was brought back to the emergency department by his mother. He was now experiencing night vision loss and his physical exam revealed bilateral cloudiness of his eyes and small whitish spots over his right iris. He was referred to a third ophthalmologist; that exam revealed bilateral xerosis, bilateral corneal edema and haze with deep stromal vessels, and a persistent right corneal abrasion. Due to their concern for the continued progression of ocular symptoms of unknown etiology, he was again sent to the emergency department. 

During the ED presentation, a detailed history was taken to better understand his illness progression and failed treatments. His mother shared that the patient was having a difficult time with the recent passing of his father. In their culture, the eldest male takes on the leadership role in the family. At eight years old, the patient was now the oldest male in their household and viewed himself as the leader, and the leader could regulate his own diet. Since his father passed away, the child had a diet consisting primarily of instant noodles from which he would pick out vegetables. His symptoms were isolated to his severe ocular symptoms, and there had been no significant fluctuations in his weight over the past several months. Given the concern for VAD, the patient was directly admitted to our institution for further evaluation of symptoms and an eye exam under anesthesia.

**Figure 1:**
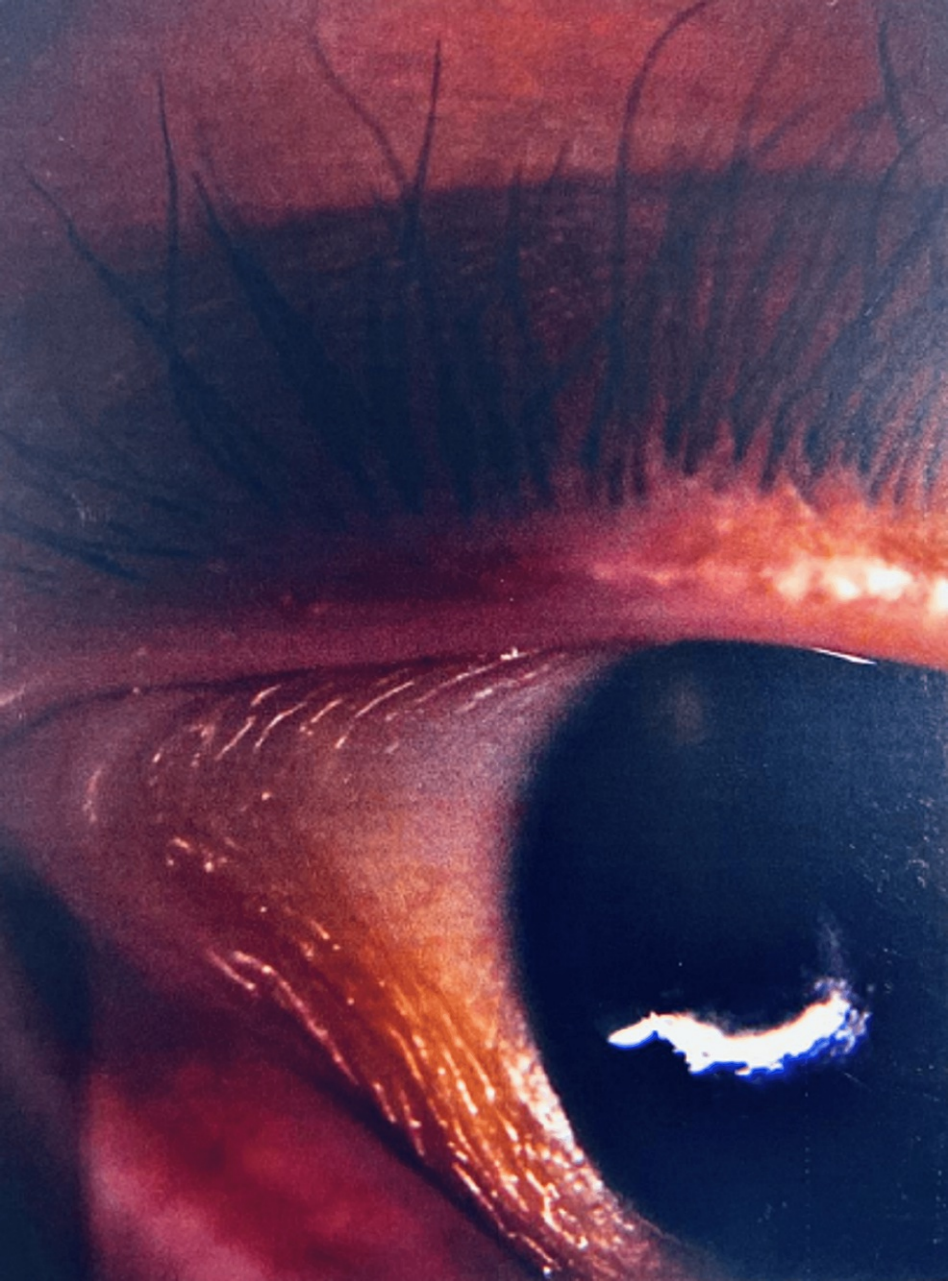
Conjunctival xerosis of the right eye.

The eye examination under anesthesia revealed bilateral conjunctival xerosis (Figure 1) with a right-sided Bitot’s spot, diffuse bilateral corneal haze with deep interstitial keratitis, and a right-sided 3mm corneal defect. Vitamin A level returned severely deficient at <2.5 ug/dL. The patient was started on 50,000 units of vitamin A supplementation daily and followed up with the ophthalmologist after discharge from the hospital. Initial follow-up visits revealed a visual acuity of 20/200 bilaterally; however, acuity slowly improved to 20/30. On his last follow-up, the patient had normal conjunctivae and cornea. 

## Discussion

VAD in the developing world is common due to food deprivation and poor availability of vitamin A-rich foods. If left untreated, VAD can result in permanent vision loss [3]. In developed countries where vitamin-enriched foods are more readily available, this deficiency is rare and is primarily related to an underlying medical condition (i.e., malabsorption) or behavioral conditions resulting in restrictive eating behaviors (i.e., anorexia and autism) [3,4,7]. The current case emphasizes the importance of initially obtaining a thorough history to determine the etiology of any patient’s chief complaints. This should always include a detailed history of diet and eating habits, including a focus on any culturally based habits. It is possible that VAD is underdiagnosed in developed countries and more common than we currently understand [8,9]. Unfortunately, our patient endured months of eye symptoms, and it was not until after two emergency department visits, two separate ophthalmologist evaluations, multiple primary care provider visits, a hospital admission, and an extensive evaluation that the correct diagnosis was made. 

Although our patient had no prior mental health or behavioral concerns, his VAD is likely related to psychological stressors resulting in a very restricted diet. The death of a parent is a distressing life event for a child; children grieve differently from adults with different emotional and behavioral challenges [10,11]. In general, cultural experience may have a mediating influence on the resolution of grief in children; there may be additional cultural dynamics that may have led to our patient’s VAD.

The fact that the patient had changed his eating patterns suggested that an intervention was needed for his grief [10]. Our patient belonged to the Hmong ethnic group, which has its origins in regions around Southeast Asia. Focus groups among the Hmong community in California define health as harmony within the family, psychological/emotional stability, and eating many types of vegetables and fruits [12]. Adults play a vital role in educating their children to eat a healthy diet [12,13]. Additionally, Hmong children report that the role of their father includes splitting cooking duties evenly [13]. It is quite possible that the death of our patient’s father affected his eating habits. Families also often resort to the convenience of fast foods and other easily accessible meals when they are too tired to cook or do not have time [12]. One would imagine that a newly widowed mother would have several challenges, including controlling all cooking duties for our patient and his siblings. Additionally, the mother must also battle the acculturation of her son’s (and possibly other children’s) dietary habits as they are constantly surrounded by American cuisine, which is typically higher in sugar, saturated fat, trans fatty acids, and sodium [14,15].

In the Hmong culture, the eldest male takes on the leadership role in the family [16]. Our patient is the eldest son, and after the death of his father, he thought of himself as the head of the household. According to his mother, it was at that point he decided to eat whatever he wanted. It would be easy for one to assume that our patient ate whatever he wanted out of defiance. These assumptions should be avoided. Although it is helpful to understand how cultural influences can contribute to the patient's environment, it is not possible to know his true intentions, his access to resources, and his level of family support or guidance. It is important that we understand the impact of cultural influences and how death, grief, diet, and familial dynamics are intimately intertwined. It is also notable that this family requested to have a Hmong interpreter present. The mother spoke some English, but it was not her first language. It is unknown whether she needed an interpreter for the several previous presentations, but this could have contributed to the delayed diagnosis. Although socioeconomic status did not seem to be a major contributor in this case, it is important to consider since it can create an additional barrier to accessing vitamin-rich foods. Ease of access to nutritional foods tends to vary based on the socioeconomic factors of the community, with low-income areas lacking nutritional resources [17]. 

## Conclusions

At such an early age, children are still learning how to regulate their own dietary habits. It is the role of the parent to support and guide their growth in this way. It is important for pediatricians to educate families on appropriate nutrition and monitor for the possibility of any deficiencies. Additionally, it is important to take a thorough history in order to identify social influences and psychological stressors that could be contributing to a patient's presentation. Further investigation is needed to better understand these cultural dynamics and how they affect behavior and nutrition.
